# Midwives’ intrapartum monitoring process and management resulting in emergency referrals in Tanzania: a qualitative study

**DOI:** 10.1186/s12884-015-0691-0

**Published:** 2015-10-08

**Authors:** Kana Shimoda, Sebalda Leshabari, Shigeko Horiuchi, Yoko Shimpuku, Junko Tashiro

**Affiliations:** Doctoral Program, St. Luke’s International University, 10-1, Akashi-cho, Chuo-ku, Tokyo, 104-0044 Japan; School of Nursing, Muhimbili University of Health and Allied Sciences, Dar es Salaam, Tanzania; St. Luke’s International University, Tokyo, Japan; St. Luke’s Birth Clinic, Tokyo, Japan

**Keywords:** Quality of care, Decision making, Clinical judgment, Childbirth, Emergency referral, Developing countries, Tanzania

## Abstract

**Background:**

In the United Republic of Tanzania, the maternal mortality ratio, and neonatal mortality rate have remained high for the last 10 years. It is well documented that many complications of pregnancy are avoidable by providing skilled midwifery care during and immediately after childbirth. However, there have been delays in providing timely and necessary obstetric interventions, most likely due to lack of proper monitoring during labor. Yet, there has been little research concerning how midwives monitor the process of childbirth. Therefore, this study aimed to describe how midwives monitored and managed the process of childbirth to achieve early consulting and timely referral to obstetricians.

**Methods:**

The design was qualitative and descriptive, using data from comprehensive semi-structured interviews of midwives. The interviews were conducted at one hospital and one health center in Dar es Salaam, Tanzania’s largest city. Eleven participants were purposively recruited and interviewed about their experiences managing complicated intrapartum cases. After the interviews, data were analyzed using content analysis.

**Results:**

Derived from the data were three activity phases: initial encounter, monitoring, and acting. During these phases, midwives noticed danger signs, identified problems, revised and confirmed initial problem identification, and organized for medical intervention or referral. The timing of taking action was different for each midwife and depended on the nature of the prolonged and obstructed labor case.

**Conclusions:**

For the majority of midwives, the processing of assessments and judgments was brief and without reflection, and only a few midwives took time to continue to monitor the labor after the initial identification of problems and before taking actions. To make a final judgment that the labor was becoming prolonged or obstructed, midwives should consider taking time to review and synthesize all their findings.

## Background

Maternal and child health is the key for progress in all the United Nation’s (UN) Millennium Development Goals (MDGs) [1]. However, compared to other countries the specific achievement toward Goals #4 and #5 have progressed less in Sub-Saharan Africa, which carries the highest burden of maternal and perinatal mortality in the world.

In Tanzania, the maternal mortality ratio (MMR) of 454 per 100,000 live births and neonatal mortality rate (NMR) 26 per 1,000 [2] have remained high for the last 10 years [3]. Tanzania’s NMR is increasing while the rate of under-five deaths has declined [2]. The main causes of maternal deaths are hemorrhage, eclampsia, obstructed labor, and infection [4]. The main cause of neonatal deaths is intrapartum asphyxia [5], which accounted for more than half of the early neonatal deaths [6, 7]. Asphyxia can be caused by obstructed/prolonged labor, which is also a risk factor for maternal complications [8] and is one of the predominant causes of maternal referral [9].

Intrapartum complications are largely avoidable through appropriate midwifery care such as adequate fetal and maternal monitoring, timely medical intervention, reducing prolonged labor, and timely referral during and immediately after childbirth [6, 7, 10, 11]. Although Tanzanian midwives are also required to diagnose, manage, and provide early referral for complications to save mother and newborn [12], most likely there have been delays in providing timely and necessary obstetric interventions due to the lack of proper monitoring during labor [6]. Supporting those findings were several studies noting Tanzanian midwives’ critically low ability to recall the danger signs of labor complications and to provide appropriate monitoring during labor and after delivery [13, 14]. However, Tanzania also has a serious shortage of human resources in the domain of perinatal care, and that might be another factor associated with the poor performance and outcomes [13].

Midwives’ monitoring outcomes also depend on referral access. The referral system in Tanzania is organized as a pyramid. The peripheral health facilities (health centers and dispensaries) are to provide basic emergency obstetric care while the district, regional, and national hospitals should provide comprehensive emergency obstetric care [15]. However, the existing maternal referral system is less effective due to lack of sufficient supplies and staff, quality skills, adequate supervision and monitoring [16], reliable means of transport, and evidence based guidelines for management of labor [17]. The improved access to obstetric care needs to go hand-in-hand with monitoring and improved quality care [18]; maternal and child health cannot be improved without a strong political decision to empower midwives and a substantial strengthening of health systems with a focus on quality of care rather than on numbers [19].

A greater understanding of how midwives monitor childbirth could reveal areas where more education would make a positive difference. Nevertheless, little study has been undertaken to explore the quality of midwives’ monitoring process of emergency care. Therefore, this study aimed to describe how midwives monitor the process of childbirth and manage it for early consulting and timely referral to obstetricians.

## Methods

### Study design

The design of this study was qualitative and descriptive with purposive sampling. Comprehensive semi-structured interviews of midwives comprised the data.

### Study setting

The research was conducted at one regional referral hospital and one health center in Dar es Salaam city, one of Tanzania’s largest cities with a population of around three million within an area of approximately 1,400 square kilometers. In Dar es Salaam the neonatal mortality rate is 28.4 per 1000 and the infant mortality rate is 70.9 per 1000 [20]. There are a total of 28 hospitals, 29 health centers and 392 dispensaries [21] employing 1,274 nurse-midwives [22].

The local supporter (SL), who was a midwife and leading researcher of maternal health and midwifery, sent a letter of request to the municipal medical officer of health seeking permission for the researchers to conduct this study at the hospitals and health centers in the city. The municipal medical officer of health provided the necessary permission for the researchers to conduct the study at one hospital and one health center. The researchers went to those settings with the interpreters to explain the components of this study before starting data collection.

### Study participants

Participants from the hospital and health center were purposively recruited. The inclusion criteria for participants were as follows: 1) midwife, 2) experienced with referral to obstetricians during the first and second stage of labor, 3) at least three years of experience as a midwife, and 4) consented to participate in the study.

Recruitment was conducted based on the principles of voluntarily participation. After explaining the purpose, methods, and ethical considerations of the study to the in-charge head nurse or charge nurse of the maternity and/or labor ward, the in-charge nurse shared it with other midwives. Only those who told their in-charge nurse of their interest participated in this study; 11 midwives indicated their interest. The researcher obtained their agreement information on the informed consent form. Prior to their interview the researcher again explained, to each participant, the purpose, methods, and ethical considerations of the study. They were informed that this study would not influence their status or relationships in their work setting. Also, they were told that they could refuse to participate even if they had previously agreed, and they could withdraw without penalty from participating even during the interview.

### Data collection

The one-on-one semi-structured interviews, which had two questions and several probing questions, focused on the midwives’ experiences of managing intrapartum complication during the first and second stage of labor. Audio-recorded interviews were conducted in a private room and in English and some in Swahili by the first author (KS). Some of the participants, who were fairly fluent in English, responded in English and other participants responded in Swahili. After finishing all the interviews, the first author transcribed the interviews from the audio-recorded data. Two clinically experienced bachelor educated midwives were the local bi-lingual (Swahili-English) interpreters during the interviews and translated the Swahili interviews into English. Moreover, one local bilingual Tanzanian PhD candidate back-translated participants’ responses from Swahili into English to correct and confirm the transcriptions and translations so as to find any missing data and as a check for accuracy. Data were collected from July to December 2013.

### Data analysis

There were a total of 15 cases of obstructed labour and prolonged labour described by participants. Of those, 11 cases were selected for the analysis of midwives’ monitoring and management process. The selected cases provided rich data and a variety of situations for a more complex picture of their experiences.

Content analysis was conducted to analyse the interview data. The first author read and reread the transcripts highlighting words, phrases and sentences that had interest and relevance to management issues. These items were then summarized and coded. The analyses included grouping similar codes to create sub-categories and similar sub-categories were integrated into categories. Finally, categories were integrated into three phases and arranged sequentially. The co-authors, who have been leading researchers of maternal health and midwifery, supervised the data analyses.

### Ethical approval

The study was accepted by the ethics review boards of 1) St. Luke’s International University, 2) Tanzanian National Institute of Medical Research and 3) Tanzania Commission for Science and Technology.

## Results

The average length of service of the 11 midwives was 12.5 years (SD = 11.3). Participants’ educational attainment varied: four had completed a certificate course, four had completed the diploma course, two had a bachelor’s degree, and one had a master’s degree.

Working at the regional referral hospital were six midwives and five worked at the health centre during the day. All 11 participants worked in the maternal and labour wards, which had a range of approximately 30 to 80 deliveries daily.

### Overview of midwives’ emergency care process for high-risk labour until referral

**Fig. 1:**
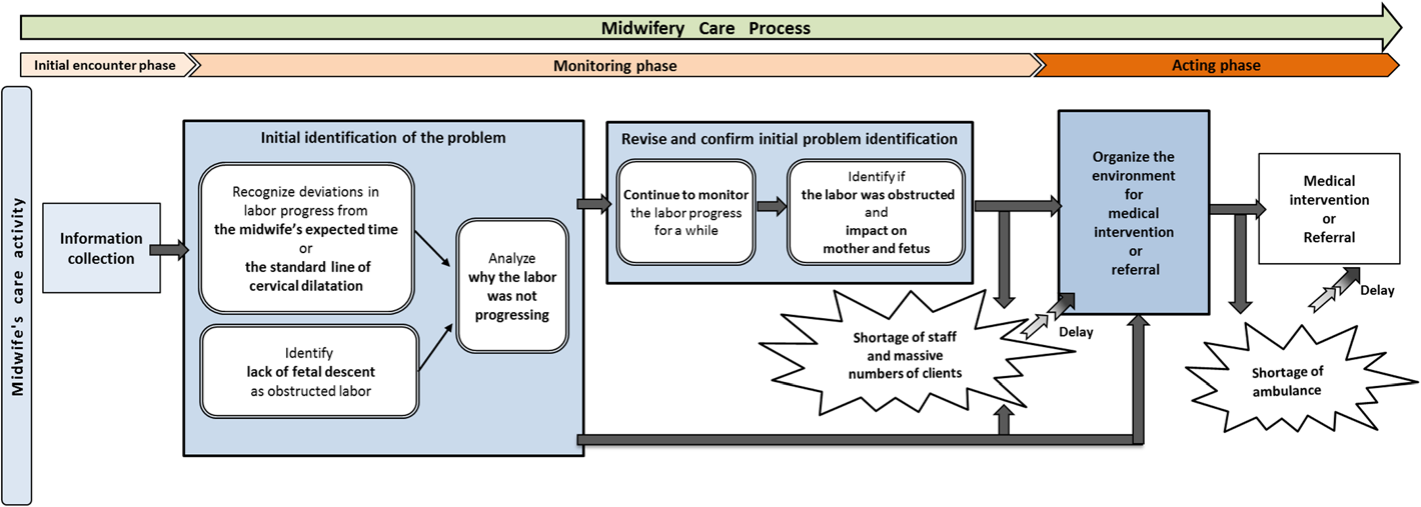
A model of three phases of midwives’ emergency care during high-risk labor

The midwives’ care process of intrapartum monitoring and management for emergency referral was derived from the data. It consisted of three phases: 1) initial encountering, 2) monitoring, and 3) acting that finally led to the referral (see Fig. 1).

The initial encounter phase occurred when midwives took charge of the mothers’ care. At this time they (a) collected information such as obstetric history, weeks of pregnancy, fetal presentation, cervical dilation, descent of the fetus, and uterine contractions and (b) observed and assessed the mother’s and fetus’s well-being and labor progress, particularly observing for danger signs.

In consideration of the danger signs, they went through the monitoring phase such as using a partograph and recalling similar past experiences to identify the problem before taking actions. In the third phase, they took actions to save the lives of the mother and fetus. They organized the environment for medical intervention or prepared for referral.

Cross cutting the phases midwives recognized several barriers impinging on their practice. These barriers were shortage of staff, massive numbers of patients, and lack of ambulances (transport) for referral.

Each phase had several interrelated care activities. However, in this study, the monitoring phase is presented in more detail to focus on the care activities of monitoring. Subcategories are indicated in italics. Likewise, quotations from the interview data are enclosed in double quotation marks and participants are anonymously indicated in the parentheses.

### Monitoring phase

The monitoring phase included three steps. The first was initial identification of the problem; next were problem revisions and confirmation of the identified problem and the last was recognition of hospital structural barriers.

### Initial problem identification

Determinants of midwives’ initial problem identification were (1) *recognized the labor progress deviated from the midwife’s expected time or deviated from the standard line of cervical dilatation*, (2) *identified lack of fetal descent as obstructed labor*, and (3) *analyzed why the labor was not progressing*.

Midwives used a partograph giving an ongoing pictorial overview of labor progress to judge the normalcy of labor progress; it included ‘alert lines’ if the labor fell outside the expected timeline. Most midwives made a diagnosis at this initial stage and would proceed to the action phase. They recognized there was a gap between some standard labor progress indicators as compared to the mother’s actual labor progress.

#### Recognize deviations in labor progress from the midwife’s expected timeline or the standard line of cervical dilation

Midwives recognized that the mother’s laboring progress was different from what they expected or was different from the standard line of normal indicated on the partograph, and that labor had been delayed.

*“I predicted, [that] after 4 h [from admission], the mother could reach 9 cm or full cervix dilated. But at the time, once I continued to monitor the progress of the mother, I could see the fundus was not descending, you can even diagnose big baby labor.” (A)*

When the midwife collected information at the time they assumed charge of the mother, they inferred how the labor would progress. They stated they normally do a ‘per vagina’ (PV) examination every four hours as routine care as indicated in the guidelines of midwifery care. They used a partograph to monitor the mother’s labor progress. Midwives had deduced that the labor would progress well based on the first PV assessment.

Yet after four hours, they noticed labor was delayed by observing the slow progress of cervical dilation or descent of the fetus; the labor progress was hours behind the standard labor progress line.

*“We saw labor [pattern], by using partograph. When we admitted this mother, she was 5 cm, and she was gravida 4. We are shown that multipara, cervix is dilated 1 cm 1 h. Now she had stayed here 4 h, and still the cervix was 5 cm, I supposed there was a problem. Because this mother was gravida 4, we are shown in 7 to 8 h must be delivery. If primipara, we can even go for 12 h, now I wanted to know why there is no progress for her”. (B)*

They recognized that the cervix should dilate 1 cm per hour if the mother was multipara. The duration of labor was also standardized. A midwife would recognize that the labor progress was abnormal by graphing a point of cervical dilation on a partograph and comparing it with the preprinted alert and action lines. When midwives found that the mother’s cervical dilation was drifting from the standard, they regarded that situation as abnormal.

*“First PV was 4 cm. After 4 h I got 5 cm. It means that the alert line [of a partograph] is [shown to the] left [of the point of 5 cm]. The mother’s dilation was going to the action line. When you see like that, you take the action by asking the doctor what to do for the mother. The mother should have dilated 1 cm per one hour.” (I)*

#### Identify lack of fetal descent as obstructed labor

The descent pattern of the fetus is one of the indications, which indicates how well the labor progresses. Midwives noticed the fetus was not progressing downward even though they could see the other findings were progressing well based on the standard.

*“I reviewed her, per vagina examination was done and the cervix was good, she was almost fully dilated. But descent of the baby was 5 over 5, so the baby was not descending though cervix was full dilated.”(D)*

Midwives recognized failure to progress even though other findings, such as cervical dilation or strong uterine contractions, were progressing normally. When midwives noticed there was no progress of the fetus descent, they suspected it would be obstructed labor through considering other findings such as a retraction ring (*Bandl’s* ring) of the uterus.

*“I assessed her and based on the assessment, I knew she was going to deliver normally. Dilation was fine, the head was moving down, but when pushing, the baby was not descending. I looked at the Bandl’s ring, then I realized it was obstructed labor.” (H)*

#### Analyze why the labor was not progressing

Midwives analyzed different possibilities for stalled labor progress. They formulated a hypothesis or diagnoses based on visible findings and their knowledge base.

*“The baby couldn’t reach the pelvic brim. So I informed the doctor the baby is still high, and is a big baby, and obstructed labor; [we are] preparing for caesarian section because the baby was big compared to the mother’s pelvis. The mother had severe contractions but she couldn’t deliver because the passage in her pelvis was too narrow”. (K)*

When midwives did a PV and noticed that the fetal descent was poor, they also examined the vaginal passage to see whether it was adequate and they considered the history of obstruction. The following midwife asked the mother about the size of her previously delivered baby to know if her pelvis would be adequate to conduct a normal vaginal delivery.

*“It [the fetus] stayed stagnant. The perineum [should be] compared to the fundus of the mother, if the perineum is too tight, the baby could not even pass there. For multigravida, you have to ask about previous kg of the baby delivered. She delivered the baby of 2.5 kg, the baby could pass there. So this could be big baby. Even the cervix was full, the head was high.” (A)*

Even if midwives thought the pelvis was within normal size limits, they might assess the fetus as too large for a normal size passage. They identified those situations as obstructed labor.

*“She progressed well but the head did not descend, even when she had full dilation and the mother had strong contractions. Presenting part was high, mother is tired and once I did head pelvis assessment I found out that she can not deliver normally. Maybe it is a big baby. So this was already obstructed labor not prolonged labor.” (G)*

This midwife was hypothesizing several different causes of poor fetal descent. One of them was a short umbilical cord and another was if the umbilical cord was around the fetus’ neck.

*“I could not only suspect a big baby, even a short cord can make the baby not descend, staying high.” (A)*

*“In this case, the dilation was okay, but with poor descent. For me in this case I thought of cord around the neck (of the fetus). Because dilation was good but descent was still high. So in my opinion, I was thinking the fetus’ neck was surrounded by cord, this can lead to this complication.” (E)*

Moreover, midwives also suspected that poor uterine contractions could be the cause of poor labor progress. The following midwife identified a ‘good’ contraction from her experience, and she identified ‘moderate’ contractions as the cause of poor labor progress.

*“She was admitted for labor pain. And when she was admitted, she was not having strong contractions. She stayed for one day, and then after continuing the assessment, I found that the mother’s contractions were poor… This is condition was prolonged labor. […] Good contractions we say are when the mother is having three strong contractions within 10 min.” (I)*

### Revise and confirm initial problem identification

When midwives noticed danger signs, they quickly tried to identify the problem and diagnose it. However, some midwives took more time and continued to monitor the labor progress or to examine other findings so as to revise and confirm the initial problem identification. They did this by continuing to monitor and then confirmed that the labor was abnormal, before making the final decision.

#### Continuing to monitor the labor progress

It took some time for midwives to decide whether they should take action or not because they expected labor to progress even though danger signs were already identified.

*“After 4 h the second PV was done at 10 pm. Cervix dilated 8 cm. Descent was still high but the mother had strong contractions. So monitoring continued. The mother was complaining of pain. I also assessed the fetal heart rate, which was starting to change. The fetal heart rate was very high. The mother had very strong contractions but descent was still poor. So after that, I gave her about half an hour to observe if descent can take place so that the mother can push well.” (E)*

Although the midwife noticed that the descending fetus was slowed, she attempted to see if a normal birth process would emerge. Because uterine contractions were strong and the cervix was already dilated 8 cm, the midwife assessed the labor as possibly progressing even though the fetal heart rate was increasing. Before midwives absolutely confirmed that they should take some actions, they tried to observe a little longer with expectations for a successful descent.

#### Confirming the problem

Before taking action, midwives finally confirmed that the labor was becoming abnormal by observing more abnormal findings, which led to a definitive diagnosis.

*“Fetal heart rate was becoming irregular by fetal scope and Doppler: 120 or 125. I thought this mother was developing a problem, because she was having strong contractions, and the fetal heart rate was becoming distressed. Her temperature was increasing. Temperature was 37.5 °C. Mother was developing maternal distress. So I started lactated ringer’s solution, and did a catheterization.” (B)*

*“When she put in the catheter, we saw some blood starting to pass. That is the sign of obstructed labor. That’s why I decided to refer immediately.” (F)*

Midwives had already suspected the labor was becoming abnormal when they noticed the first danger signs as noted in the initial identification. Then the midwife saw the clear presence of blood in the urine and took immediate action. However, in similar situations some midwives took earlier actions instead of taking additional time to confirm the labor, as the status of the mother and fetus was deteriorating.

### Shortage of staff and massive numbers of clients

During the interviews, midwives mentioned that sometimes they had cases with serious complications, which could not be referred on time. They said in those cases there were barriers commonly resulting from *staff shortages* and *massive numbers of clients*.

*“First of all [I] informed our in-charge nurse. In-charge come and saw this case, the in-charge said inform the doctor because there was no progress. Doctor said, I’m coming but she was still in the surgical theatre. She was doing a caesarian section. After 4 h, again, [after the] third PV, I saw the progress was not good. There was no progress. The doctor came and found that the fetal heart rate was also not good. It was around 120. At the third time calling, we planned for a caesarian section. […] The Doctor was [still] busy. Even if she would come to examine, she could not take the patient to the surgical theatre because there was already one [patient] in the theatre. There are so many patients. We even have two theatres, but sometimes they are crowded.” (B)*

When the midwife conducted the second PV examination, she had already noticed the laboring progress was poor and she informed her colleague. However, in spite of the colleague informing the doctor, the doctor was too busy performing a caesarian section to attend to another patient. Because there were many patients and not enough staff or surgical beds, the midwife had to wait to decide what action to take next. The following midwife also mentioned it was sometimes difficult to take appropriate action on time because of shortage of staff.

*“I was running here and there to look for ringer’s solution and catheters. This is our cry because even if I have few patients I am always busy chasing after the pharmacist to provide me with catheters, and ringer’s solution. But they promised to bring them, but remember, patients were waiting for my service. If we would have enough staff and supplies soon after identifying a problem, you can perform your duties then call the doctor for discussing the identified problem and make a decision if we can do caesarian or refer patient soon according to the problem.” (F)*

### Shortage of ambulances

Lack of available ambulances was another barrier, which caused the delay of medical interventions or referrals. At the study sites, there were only two ambulances assigned to four hospitals. Those ambulances were not only used for the patients from the maternity and labor wards, but also for all the other patients. Therefore the problem was often no ambulance for the immediate transport of the patient.

*“It was during the time when the doctors were not around and the anesthetist also had already left. These are challenges we normally face when we have two (problem) cases at the same time, and the ambulance has already left to take one of the cases to the hospital. The patient was sent to hospital “A” and delivered her baby but the baby’s Apgar score was low. But I think if by the time we called it as an emergency and the ambulance had been available, the baby would have scored well.” (F)*

The other problem with the ambulances was related to shortage of staff. Even if there had been an ambulance available at the time, sometimes there would be only one driver who might not be available.

*“If the ambulance comes late, we have to stay with baby and the mother. While we are waiting for the ambulance, the doctor is here together with patient until ambulance comes. Sometimes we have only one driver, so he uses only one ambulance.” (K)*

## Discussion

### Premature decisions without synthesizing the findings

In each case of difficult labor, as described by the midwives, there came the time when midwives judged it would be too risky to continue monitoring labor and it was time to call a doctor. However, the timing to take action differed depending on the case. After identifying the gap in expected labor progress, two patterns emerged: continuing to monitor or taking action.

The majority of midwives decided to take action immediately after they noticed danger signs and identified the symptoms as determinants of intrapartum complications. They reasoned those findings would either get worse later or that it was already at the emergency stage. WHO [23] recommended that it is essential to consider the history, the physical examination and the partograph to identify problems of prolonged and obstructed labor; it can only happen with much thought. Mouni [24] also indicated that midwives must have sufficient information about the problem before applying any judgment; it is a key for effective decision-making. Unlike a nearly instantaneous abnormality, prolonged and obstructed labor can only be identified after much observation and consideration. Even though the midwives gathered a large amount of data and recognized the basic danger signs, which are determinants of complications, it was possible that they failed to adequately synthesize the important findings.

There were some other factors that could lead midwives’ arriving at premature decisions. Midwives identified prolonged and obstructed labor using the partograph thus leaving the partograph information as the final decision-making factor. When the cervix was not dilating along the normal line of the partograph, they identified the labor as abnormal. Maaloe [25] indicated that although the partograph is simple and clear to use for classifying data, a bias might occur because other information was missing. To rely too much on the partograph as the standard indicator may have obfuscated midwives’ own assessment and judgment.

### Necessity of synthesizing and deliberating before a final decision

Midwives, who did not inform or refer to a doctor immediately, took their time to continue to monitor the labor after identifying initial problems. In such a reflective process, midwives go through several iterations of observation, assessment, judgment, and action; each cycle informing the next. Thompson [26] introduced the information-processing model in his study, and according to the model, interpretation of the data, which is gathered during the initial stage would be classified as to its significance to include, refute or not contribute to the initial hypotheses. Likewise, Mong-Chue [27] described that reasoning is another way of figuring or ordering the information, and it includes the process of judging the relation between premises and conclusions. However, the assumptions should be called into question whether it is false or true. Therefore, to ensure adequate reasoning one must obtain reliable data, look at problems from a different point of view, and pay attention to other contexts [27]. It might be reasoned that the midwives expected the labor to progress. Instead of dealing with danger signs immediately, they tried to determine if they could spend more time to monitor the labor. They had a positive prediction that the labor could progress because the mother had strong uterine contractions. To make a final judgment that the labor was becoming abnormal, they would have synthesized all other findings, including their knowledge and experience.

### Possible factors influencing midwives’ decision-making

#### Overload of patients and lack of staff and hospitals

Midwives’ working conditions may have interfered with their ability to make appropriate monitoring and action decisions. They reported that only three to four midwives were available to follow 30 to 40 deliveries nightly and there was an average of 60 to 80 deliveries daily. Under such difficult conditions, they may not have been able to monitor fetal heart rates every half hour and would tend to identify fetal abnormalities based on only a few intermittent auscultations. Likewise, under those circumstances it was possible that they could not monitor uterine contractions on schedule because of the large patient load they were attempting to monitor. Therefore, it may be difficult for midwives to determine and sort out which mothers were in need of a referral and that would also explain the tendency to just rely on the partograph as the main time saving cue. Because of the shortage of health institutions, if all such laboring mothers, who were believed to be abnormal, were referred, the busy referral hospital would become even busier. As a result, the emergency referrals could create a vicious cycle and mothers who really needed referral would be delayed.

#### Dysfunctional electricity, supplies and health systems

Midwives’ decision-making was also negatively influenced by such factors as staff shortage, stress levels, and patient turnover. These were the same factors found in another study as well [24]. Midwives’ competency was challenged through not having enough appropriate equipment, health systems, and supplies to enable their critical thinking. This finding was also consistent with a comprehensive review of maternity care in low-resourced countries [28]. Due to huge inconsistencies between ideal textbook methods and the limitations of reality, midwives might not have been able to develop their competency.

The timing of the midwives decisions to refer was also dependent on various environment factors. Even though there were operational systems for caesarian sections and blood transfusion systems at their own hospital, they could not implement a caesarian section because of external factors such as electrical outages and no available physicians. Although midwives were often timely in noticing danger signs and they accurately predicted that the mother’s condition would be worse, the external conditions created a referral barrier. Developing countries have a long history of chronic shortages of supplies and facilities [8, 29]. This study also had similar results as previous studies, and although none of these conditions are new, they are significant problems and must not be ignored. Therefore, to further support midwives, policies to empower midwives and to augment health systems are necessary [19].

Those mothers who were referred could have had surgical procedures at their own hospital if circumstances had been normal. Also, the time necessary to save the baby carries different prognostic implications. Skilled, speedy and quality care will only work when the necessary functioning equipment, functioning health systems, including transport and referral facilities during emergencies, are operating smoothly [23]. Even midwife’s care that is of a high standard cannot maintain itself in the absence of supporting systems. When considering quality midwifery care, it is essential to take into consideration the quality of the working environment.

### Necessity of continuing education for midwives

Midwives’ care was appropriate to identify the intrapartum problem and to intervene, however, there was also room for better quality care. Some midwives used decision making characterized by one main factor or criterion [30], but even though it did not suit the monitoring labor process carrying out an overall labor assessment is important. Styles et al. indicated that clinical practice has always embraced a systematic and analytical approach to decision-making [31]. To develop such skills, it might be insufficient to learn only basic midwifery knowledge and skills during pre-service education; this means in-service education and memorable experiences are crucial. According to Styles et al., recent and/or dramatic memories of clinical incidents can be influential when midwives make clinical judgments [31]. Although basic midwifery education constitutes the majority of midwifery basic and core competencies [32], midwives may not be able to apply the standards and basic knowledge for those unpredictable aberrant cases. Also, relying solely on standard indicators might not help to gain reflective experiences, which influences their next decision-making.

Although there are pre-register education programs in Tanzania [12], constant in-service education is not formally instituted at any hospital in Tanzania. It cannot be possible to enhance midwives’ capabilities without continuing education. In previous studies [33, 34], researchers indicated that the midwife’s own personal experiences and actions should be drawn with retrospection and reflection to access to tacit knowledge. This can assist to gain expertise in making clinical judgments in midwives’ professional practice. Nakielski et al. [35] also reported that constant reflection-on-action to assess as to whether a judgment or decision was right in retrospect and reflection will serve as a source of encouragement. If there is an opportunity to reflect on midwife’s individual experience and to continually share with other midwives in daily practice, for example in case conferences, their skills of reflection and cyclical decision-making may be developed. Sharing and reflecting with midwives from various backgrounds provide midwives with a golden opportunity to learn, enhance skills, and build trusting relationships. Likewise, it might be necessary to empower midwives to clarify what midwives can do and what they should do to enhance their practice, and to formulate solution for themselves through shared experiences with each other in clinical practice.

Additionally, to fill in the gaps between the textbook ideal and the stark reality, it is obvious to note that the most crucial issue is improvement in midwives’ working conditions. Management of care is one of the standards of the nursing education programs of Tanzania Nurse and Midwives Council [12]. According to the standard, not only to participate actively in care provision, but also to accept responsibility for the effective and efficient management of the care and practice within a safe environment is essential for the nursing role [36]. To avoid unnecessary interventions and double work, the appropriate environment is critical. It is essential that midwifery practice include not just competent care for the mother and fetus or baby, it must also include a perspective of management of organization, team collaboration, risk-control ability and appropriate facilities.

### Strength and limitation of this study

The strength of this study is that it used midwives’ narratives to describe their monitoring and management process. It is one of the few studies that provided a focus on qualitative data, documenting midwives’ actual thinking and their difficulties.

There are some limitations of this study. Participant observation might have provided additional abundant data for understanding midwives’ working conditions. Likewise, because the context of the interviews was focused on only the first and second stage of labor to understand the midwife’s labor monitoring process, other complicated cases may have been omitted. Finally, cross cultural interviewing and translation always runs the risk of missing or distorting information. Attempts were made to correct for that possibility through careful interviewing, back translation, and supervised coding.

### Further research

Further research is needed to understand more about the quality of midwifery care during childbirth. It should be conducted under a variety of different conditions such as lower level health institutions, rural areas and private hospitals. A quantitative study with a large sample size, to eliminate participant’s bias and disparity between urban and rural area could be conducted and could include patient outcomes. Determining what ‘competent’ midwife means is quite difficult and problematic. To explore what is competent and how it should be in each country, requires further study considering not only midwives’ care but also the working condition and environment.

## Conclusion

Midwives described their midwifery care process when confronted with a pregnant woman whose labor was not progressing and may need referral. The timing of taking action was different depending on the midwife and available resources. The majority of midwives decided to take action immediately after they noticed danger signs and only few midwives took time to continue to monitor the labor after the initial identification of problems before taking actions. To make a final judgment that the labor was becoming prolonged or obstructed, it might be necessary for midwives to synthesize all their findings not by a superficial and brief assessment but by a more reflective decision making process. To acquire such a skill, may be beyond the basic midwifery knowledge and skills in pre-service education; ongoing continuing in-service education is crucial.

## Abbreviations

MDGs: Millennium Development Goals
MMR: Maternal mortality ratio
NMR: Neonatal mortality rate
PV: Per vagina examination
UN: United Nations
WHO: World Health Organization
